# Dynamics associated with the efflux pumps in biofilms and persister cells of *Cryptococcus neoformans* and *Cryptococcus gattii*

**DOI:** 10.1128/spectrum.00203-26

**Published:** 2026-07-13

**Authors:** Gabriel Rezende Pimenta, Marcos William de Lima Gualque, Maria Júlia Siqueto, Samanta de Matos Silva, Luis Otávio Regasini, Maria José Soares Mendes Giannini, Ana Marisa Fusco Almeida

**Affiliations:** 1 Laboratory of Clinical Mycology - Department of Clinical Analysis, School of Pharmaceutical Sciences, São Paulo State University (UNESP) 153998, Araraquara, São Paulo, Brazil; 2 Nucleus of Proteomics and Monoclonal Antibodies - Department of Clinical Analysis, School of Pharmaceutical Sciences, São Paulo State University (UNESP) 153998, Araraquara, São Paulo, Brazil; 3 Department of Chemistry and Environmental Sciences, Institute of Biosciences, Humanities and Exact Sciences, São Paulo State University (UNESP) 28108, São José do Rio Preto, Brazil; Universita degli Studi del Molise, Campobasso, Italy

**Keywords:** *Cryptococcus*, biofilm, resistance, efflux pump, persister cells, therapy

## Abstract

Cryptococcosis is a systemic mycosis affecting immunocompromised individuals (mainly *C. neoformans*) and immunocompetent hosts (mainly *C. gattii*). Effective treatment remains challenging due to antifungal resistance, driven by efflux pumps, target enzyme mutations, biofilm formation, and persister cells. This study evaluated efflux pump gene expression and its role in biofilm development and resistance in persister cells of both species, as well as the effects of combined treatments—fluconazole + FK506 and fluconazole + octyl protocatechuate—on biofilms. Materials and Methods included microscopy, biofilm characterization during formation, treatment assays, induction of persister cells, and relative quantification of efflux pump genes *AFR1*, *AFR2*, *AFR3*, and *MDR1*. Biofilms of both species were robust, with increased extracellular matrix linked to efflux pump overexpression. Combined treatments reduced biofilm growth and extracellular matrix production while downregulating *AFR1*, *AFR2*, *AFR3*, and *MDR1*, highlighting a link between efflux pumps and matrix export. Persister cells displayed low metabolic activity under amphotericin B treatment, followed by slow regrowth and reactivation of efflux pumps. These findings demonstrate multifaceted roles of efflux pumps in antifungal resistance and biofilm development, confirm the presence of persister cells in *C. neoformans* and *C. gattii*, and suggest novel therapeutic strategies for cryptococcosis.

**IMPORTANCE:**

Cryptococcosis is a fungal infection with mortality exceeding 75%, predominantly affecting immunocompromised individuals—including transplant recipients and people living with HIV—but also capable of infecting healthy people. Treatments are lengthy, toxic, and increasingly ineffective due to antifungal resistance. Two major contributors to therapeutic failure are biofilms—protective fungal communities encased in a self-produced matrix—and persister cells, a dormant subpopulation that survives lethal drug concentrations and reactivates infection once treatment stops. This study reveals that efflux pumps—proteins that expel drugs from fungal cells—are not only resistance mechanisms but also active contributors to biofilm construction. Critically, persister cells reactivate their efflux pumps upon recovery, providing a biological explanation for infection recurrence. Two drug combinations—fluconazole + FK506 and fluconazole + octyl protocatechuate—synergistically disrupted biofilms and reduced persister cell viability while potentially lowering drug doses and toxicity, pointing toward safer, more effective strategies for cryptococcosis.

## INTRODUCTION

*Cryptococcus neoformans* and *Cryptococcus gattii* constitute the primary species complexes of the genus *Cryptococcus* and are recognized as pathogenic fungi capable of causing cryptococcosis, a disease that can disseminate systemically within the host. Cryptococcosis is among the four most prevalent fungal infections worldwide, with an estimated 194,000 annual cases and a mortality rate exceeding 75% according to global epidemiological data. Multicenter studies have highlighted its growing incidence and classified it as one of the fungal infections of the greatest clinical relevance (1, 2). Species of *Cryptococcus* are geographically widespread, with *C. neoformans* being more prevalent in urban environments and *C. gattii* in wooded regions in addition to their capacity to adapt to diverse regions and hosts (immunocompromised—mainly *C. neoformans*; immunocompetent—mainly *C. gattii*) (3).

Cryptococcosis is managed with an induction therapy regimen consisting of a combination of amphotericin B (AMB) and 5-fluorocytosine (5-FC) for 4 weeks, followed by an 8-week maintenance phase and a consolidation phase lasting 6–12 months with fluconazole (FLC) (1). Despite its efficacy, antifungal treatment presents significant limitations. AMB is associated with nephrotoxicity, while 5-FC can induce hepatotoxicity. Moreover, clinical isolates resistant to FLC have been reported. The rising incidence of invasive mycoses and the emergence of antifungal resistance are matters of concern. Antifungal resistance compromises treatment efficacy, particularly due to the prolonged use of these agents, a particularly critical issue in immunocompromised patients (4).

Resistance arises through several mechanisms: *ERG11* mutations, the activation of alternative metabolic pathways, the overexpression of efflux pumps (primarily ABC and MFS transporters), biofilm formation, and the emergence of persister cells (5–9). Biofilms are structured communities encased in a self-produced extracellular polymeric matrix (EPM), conferring resistance up to 1,000-fold higher than planktonic cells, with elevated efflux pump activity (7, 8). Persister cells are a dormant biofilm subpopulation capable of surviving lethal antifungal concentrations and reinitiating infection upon stress removal (9).

Among the therapeutic alternatives under investigation, octyl protocatechuate, a phenolic compound, acts synergistically with azoles and exhibits antifungal and antibiofilm properties (10–12). FK506 (tacrolimus) has shown potential to reverse resistance when combined with conventional antifungals by reducing efflux pump-mediated drug extrusion (13). Given the urgent need for improved strategies, this study aimed to evaluate efflux pump gene expression and its role in biofilm development and persister cell resistance in *Cryptococcus neoformans* and *Cryptococcus gattii* and to assess the efficacy of fluconazole + FK506 and fluconazole + octyl protocatechuate combination treatments (1, 2, 4, 8).

## MATERIALS AND METHODS

### Microorganism culture

*Cryptococcus neoformans* (ATCC 90112) and *Cryptococcus gattii* (ATCC 56990) were obtained from the Clinical Mycology Laboratory, School of Pharmaceutical Sciences, UNESP Araraquara, maintained on Sabouraud Dextrose Agar at 37°C, and subcultured every 24 h prior to experiments.

### Biofilm formation

Biofilm formation followed Martinez and Casadevall (14) with modifications by Gualque et al. (15). Cultures in Sabouraud Dextrose Broth (150 rpm, 24 h) were harvested, washed twice with 0.85% saline, and resuspended at 1 × 10⁷ cells/mL. Suspensions (100 μL) were added to polystyrene 96-well plates (TPP) and incubated at 37°C for 2 h for initial adhesion. After washing with PBS and replacing with RPMI-1640, biofilms were incubated for 12 h (early-stage), 24 h (intermediate-stage), or 48 h (mature). Negative controls contained medium only; positive controls included medium with yeast inoculum. All assays were performed in triplicate.

### Multiparametric multiphoton microscopy

Suspensions were added to 24-well plates with glass coverslips, incubated at 37°C, stained with 100 μg/mL Calcofluor White and 100 μg/mL Fluorescein Diacetate for 60 min in the dark, and visualized on a Carl Zeiss LSM 780/LSM 7MP (FMRP-USP; 40 × oil immersion; FIJI 2.14.0).

### Biofilm extracellular matrix extraction

Biofilms were resuspended in sterile water, vortexed, sonicated for 20 min, and centrifuged at 1,500 × *g* for 20 min; the supernatant was used for downstream analyses (16).

### Biofilm characterization

SEM was performed after glutaraldehyde fixation (2.5%, 4°C, 1 h), graded ethanol dehydration, and gold sputter-coating on a Jeol JSM-6610LV (UNESP Araraquara). Biomass was quantified with 0.1% crystal violet (570 nm) (17), extracellular matrix with 1% safranin (492 nm) (18), metabolic activity by XTT assay (490 nm) (14), extracellular polysaccharides by phenol-sulfuric acid method with glucose standard (485 nm) (19), proteins by Bradford with BSA standard (595 nm) (20, 21), ergosterol by KOH-methanol saponification and hexane extraction (282 nm) (22), eDNA by phenol:chloroform:isoamyl alcohol extraction after RNase treatment (260 nm) (23), and eRNA using RNeasy Plant Mini Kit (Qiagen) with NanoDrop (24).

### RT-qPCR gene expression

Target genes *AFR1, AFR2, AFR3, and MDR1* were quantified by RT-qPCR. Total RNA was extracted with RNeasy Plant Mini Kit (Qiagen), treated with DNase I (Sigma-Aldrich), and integrity verified on an Agilent 2100 Bioanalyzer. cDNA was synthesized using Universal cDNA Synthesis Kit II (Exiqon). qPCR was performed with Maxima SYBR Green/ROX Master Mix (2×; Thermo Scientific) on an Applied Biosystems 7500 cycler. Primers are listed in Table 1. Data were analyzed using the 2^⁻ΔΔCt^ method with β-tubulin as the endogenous reference; gene expression controls for biofilms: untreated planktonic samples; for treated conditions: respective untreated controls. All experiments were performed in three independent biological replicates. Data were expressed as mean ± SD.

**TABLE 1:** List of primers used for RT-qPCR analysis

	*C. neoformans*	
	*Fw*	*Rv*
*βTUB* (CNAG_04948)	ACCCTGTGCTCGATGTTCTG	CCAAGACCGGAACCAGTACC
*AFR1* (CNAG_00730)	GAGTTCACCAGCTCGGAAAG	CACCTTCGATCACACCAATG
*AFR2* (CNAG_00869)	GGTTCCGACTACATGGCTGT	GAGTTCACCAGCTCGGAAAG
*AFR3* (CNAG_06909)	GCCCATAGCACTAGGAGTCG	ATGTCATCCTGCGAATAGCC
*MDR1* (CNAG_00796)	CTCTTGATCACATCGCGAAA	ACCGACAATCTTGCTCTGCT

	*C. gattii*	
*βTUB* (CNBG_3361)	GAAAGGCTTTCGTGCACTGG	AGATTTCGTCATCCGCTCCC
*AFR1* (CNBG_1200)	GCCACTCAGCATGACGAACA	GACCGACTTATCGCCCTCTTC
*AFR2* (CNBG_6088)	CTGATTTTCTGCGGTGTATTGG	AACGGCGAAACACGATACATAA
*AFR3* (CNAG_06909)	TCGGCTATTCGCAGGATGGC	GGTCCTCGATGTAAGCTGGG
*MDR1* (CNBG_1138)	CAAGCCGGAAACGTCTTCA	TGGAAGCGGCCGATGA

### Treatment assays

The octyl protocatechuic acid derivative was provided (Dr. Luis Otávio Regasini) through the Biota (FAPESP) and Sisbiota (CNPq) projects (UNESP Araraquara). Fluconazole (FLC), FK506, and octyl 3,4-dihydroxybenzoate (OCT) were tested alone and in combination (FLC + FK506; FLC + OCT) at 0.421–1,728 μg/mL in biofilm assays (2, 12, 24, 48 h) and at 0.125–512 μg/mL for planktonic cells per CLSI M27-A3 (25). Minimum inhibitory concentration (MIC) was defined as the concentration causing 50% reduction in metabolic activity. Synergy was assessed by fractional inhibitory concentration index (FICI): synergistic if FICI <0.5, indifferent if 0.5–4.0, and antagonistic if >4.0 (25).

### Recovery, induction, and treatment of persister cells

Mature 48 h biofilms were treated with 100 μg/mL AMB at 37°C for 48 h (after, XTT assay). After washing, cells were resuspended in PBS, serially diluted, plated on Sabouraud Dextrose agar, and incubated at 37°C for up to 10 days for CFU counting. Cells surviving AMB were considered persister cells (26, 27). MIC and MFC assays used AMB at 100–800 μg/mL and FLC, FK506, and OCT at 2–4,096 μg/mL by resazurin assay (25, 28). All in triplicate.

### Statistical analysis

Data were analyzed using unpaired *t*-test with normality assessment and two-way ANOVA with Tukey’s post hoc test (GraphPad Prism 8.0). Statistical significance was defined as *P* < 0.05.

## RESULTS

### Biofilm characterization of *Cryptococcus neoformans* (ATCC 90112) and *Cryptococcus gattii* (ATCC 56990)

#### Scanning electron microscopy of biofilms

**Fig 1:**
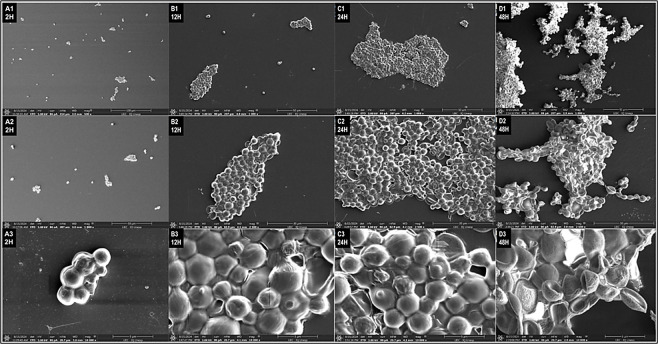
Scanning electron microscopy (SEM) of the biofilm of *C. neoformans* ATCC 90112. (A1) 2 h—Pre-adhesion at 500×. (A2) 2 h at 1,000×. (A3) 2 h at 10,000×. (B1) 12 h—Initial adhesion at 1,000×. (B2) 12 h at 2,500×. (B3) 12 h at 10,000×. (C1) 24 h—Intermediate adhesion at 1,000×. (C2) 24 h at 2,500×. (C3) 24 h at 10,000×. (D1) 48 h—Maturation at 1,000×. (D2) 48 h at 2,500×. (D3) 48 h at 10,000×.

**Fig 2:**
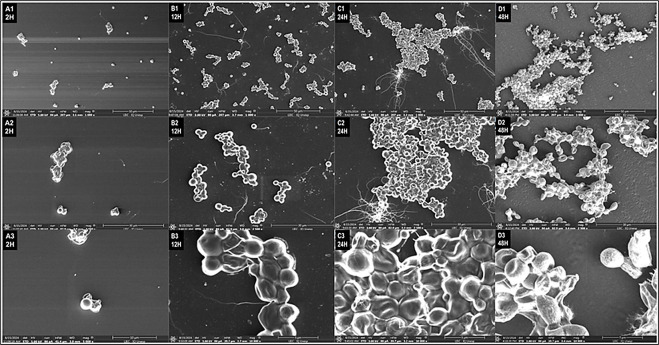
SEM of *C. gattii* ATCC 56990 biofilm. (A1) 2 h—Pre-adhesion at 1,000×. (A2) 2 h at 2,500×. (A3) 2 h at 5,000×. (B1) 12 h—Initial adhesion at 1,000×. (B2) 12 h at 2,500×. (B3) 12 h at 10,000×. (C1) 24 h—Intermediate adhesion at 1,000×. (C2) 24 h at 2,500×. (C3) 24 h at 10,000×. (D1) 48 h—Maturation at 1,000×. (D2) 48 h at 2,500×. (D3) 48 h at 10,000×.

At the 2-h stage, microscopy (Fig. 1 and 2) revealed sparse yeasts with little evidence of aggregation. The yeasts were mostly in a planktonic state, with minimal production of microcolonies. The morphology was comparable between *C. neoformans* and *C. gattii*, suggesting that both organisms follow a similar initial pattern of pre-adhesion to the surface. By 12 h, in both species, an increase in cell density was observed, with more visible clusters of cells forming a more organized structure, indicative of microcolony formation and extracellular matrix production. After 24 and 48 h, biofilms of both strains appeared more firmly attached, with a more prominent extracellular matrix. Adhesion filaments were also observed in the 24- and 48-h biofilms of both species.

### Kinetics of biofilm formation without treatment

During the first 2 h, *C. neoformans* and *C. gattii* cells showed initial adhesion with low biomass, reduced absorbance, minimal extracellular content, and low metabolic activity. By 12 h, biomass and absorbance increased, microcolonies emerged, and extracellular matrix production began, accompanied by higher metabolic activity. Between 24 and 48 h, biofilms reached maturity, with maximal biomass, structured clusters, high absorbance, and peak metabolic activity. From 24 h onward, parameters stabilized, indicating completed maturation. Both species behaved similarly although *C. gattii* showed slightly higher values.

### Quantification of target gene expression in biofilms (pre-treatment)

**Fig 3:**
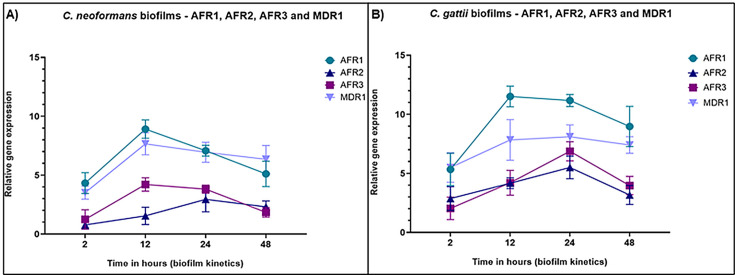
(**A**) Efflux pump gene expression in the biofilm form of *C. neoformans* ATCC 90112. AFR1 (green); AFR2 (dark blue); AFR3 (purple); MDR1 (light blue). (**B**) Efflux pump gene expression in *C. gattii* ATCC 56990 biofilm. Gene expression control: planktonic samples.

Regarding biofilm analyses, substantially similar patterns were observed between *C. neoformans* and *C. gattii*. The expression of *AFR1* and *MDR1* increased up to 12 h, reaching a peak, and then progressively declined until 48 h (Fig. 3). The *AFR1* gene exhibited the highest expression among all genes, followed by *MDR1*. The expression of *AFR2* and *AFR3* was lower under all conditions but followed a similar pattern, with an initial increase followed by a decrease or stabilization after 24 h. These results suggest that *AFR1* and *MDR1* play a significant role during the early stages of biofilm development in *C. neoformans*, facilitating initial adaptation during formation. The subsequent decrease in expression after the peak may indicate adaptive regulation as the biofilm matures and stabilizes extracellular matrix levels and metabolic activity.

### Treatment of planktonic and biofilm conditions with selected compounds

The MIC of fluconazole indicates limited efficacy when used alone, particularly against *C. gattii*. The MICs of FK506 and the octyl derivative for *C. neoformans* and *C. gattii* suggest moderate efficacy. The results indicate that both FK506 and the octyl derivative potentiated the action of fluconazole, reducing the amount required to inhibit fungal growth. *C. gattii* exhibited slightly lower susceptibility to both individual compounds and their combinations compared to *C. neoformans* (Table 2). Both combinations (fluconazole + FK506 and fluconazole + octyl derivative) demonstrated significant synergy (FICI < 0.5) against *C. neoformans* and *C. gattii*, indicating that these combinations are more effective than the compounds used individually (Table 3).

**TABLE 2:** Minimum inhibitory concentration (MIC) for inhibiting fungal growth in the planktonic form

Minimum inhibitory concentration (MIC)—planktonic form					
Strain	Fluconazole	FK506	Octyl	Fluconazole + FK506	Fluconazole + octyl
ATCC *Cn* 90112	2.0 µg/mL	1.0 µg/mL	1.0 µg/mL	0.250 µg/mL	0.125 µg/mL
ATCC *Cg* 56990	4.0 µg/mL	2 µg/mL	2.0 µg/mL	0.500 µg/mL	0.250 µg/mL

**TABLE 3:** Fractional inhibitory concentration index (FICI)—planktonic form

Fractional inhibitory concentration index (FICI)—planktonic form		
Strain	Fluconazole + FK506	Fluconazole + octyl
ATCC *Cn* 90112	0.375	0.187
ATCC *Cg* 56990	0.375	0.187

Regarding biofilms, MICs increased with incubation time in both species, reflecting enhanced resistance over time. *C. gattii* generally required higher concentrations than *C. neoformans* (Tables 4 and 5). FICI values were below 0.5 in all tested conditions, confirming synergy; the synergistic effect was consistently stronger with the octyl derivative.

**TABLE 4:** Minimum inhibitory concentration (MIC) for inhibiting fungal growth at 2, 12, 24, and 48 h of biofilm development

Minimum inhibitory concentration (MIC)—biofilm (2 h)					
Strain	Fluconazole	FK506	Octyl	Fluconazole + FK506	Fluconazole + octyl
ATCC *Cn* 90112	6.75 µg/mL	3.37 µg/mL	3.37 µg/mL	0.842 µg/mL	0.421 µg/mL
ATCC *Cg* 56990	13.5 µg/mL	6.75 µg/mL	6.75 µg/mL	1.68 µg/mL	0.842 µg/mL

Minimum inhibitory concentration (MIC)—biofilm (12 h)					
ATCC *Cn* 90112	108 µg/mL	27 µg/mL	27 µg/mL	3.37 µg/mL	1.68 µg/mL
ATCC *Cg* 56990	216 µg/mL	54 µg/mL	54 µg/mL	6.75 µg/mL	3.37 µg/mL

Minimum inhibitory concentration (MIC)—biofilm (24 h)					
ATCC *Cn* 90112	432 µg/mL	108 µg/mL	108 µg/mL	6.75 µg/mL	3.37 µg/mL
ATCC *Cg* 56990	864 µg/mL	216 µg/mL	216 µg/mL	13.5 µg/mL	6.75 µg/mL

Minimum inhibitory concentration (MIC)—biofilm (48 h)					
ATCC *Cn* 90112	432 µg/mL	108 µg/mL	108 µg/mL	6.75 µg/mL	3.37 µg/mL
ATCC *Cg* 56990	864 µg/mL	216 µg/mL	216 µg/mL	13.5 µg/mL	6.75 µg/mL

**TABLE 5:** Fractional inhibitory concentration index (FICI)—biofilm (all time points)

Fractional inhibitory concentration index (FICI)—biofilm (2 h)		
Strain	Fluconazole + FK506	Fluconazole + octyl
ATCC *Cn* 90112	0.374	0.373
ATCC *Cg* 56990	0.187	0.187

Fractional inhibitory concentration index (FICI)—biofilm (12 h)		
ATCC *Cn* 90112	0.156	0.077
ATCC *Cg* 56990	0.156	0.078

Fractional inhibitory concentration index (FICI)—biofilm (24 h)		
ATCC *Cn* 90112	0.078	0.039
ATCC *Cg* 56990	0.041	0.023

Fractional inhibitory concentration index (FICI)—biofilm (48 h)		
ATCC *Cn* 90112	0.078	0.039
ATCC *Cg* 56990	0.041	0.023

### Multiphoton microscopy of biofilms

**Fig 4:**
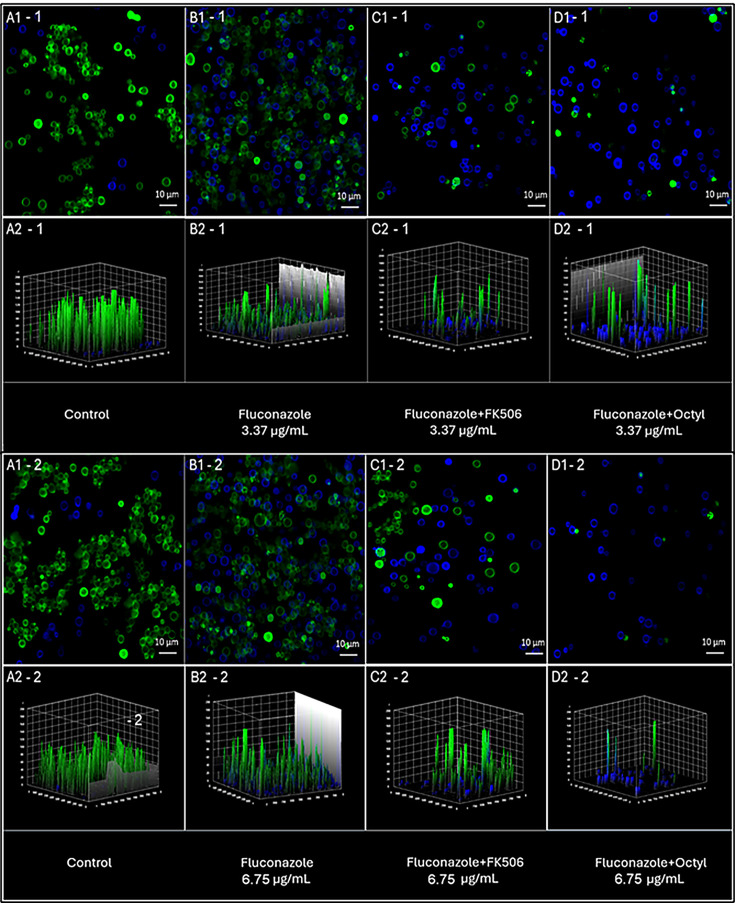
Multiphoton microscopy of *C. neoformans* ATCC 90112 (A1–D2/1) and *C. gattii* ATCC 56990 (A1–D2/2) biofilms treated with the MIC of the fluconazole + octyl derivative combination. Surface plots shown in A2–D2/1 and A2–D2/2. Calcofluor White (blue) stains the fungal cell wall; Fluorescein Diacetate (green) stains viable cells. Control: mature biofilm without treatment.

Regarding *C. neoformans* biofilms (Fig. 4A1 through D2/1), the control exhibited a high density of viable cells, with intense green fluorescence and few signs of dead cells, indicating a thick and healthy biofilm. Treatment with fluconazole alone at 3.37 μg/mL caused a slight reduction in cell viability, reflected by decreased green intensity and lower density in the three-dimensional structure. When fluconazole was combined with FK506 at the same concentration, further reduction of viable cells was observed, as evidenced by reduced green fluorescence and an even lower cell density in the volumetric profile. Finally, the combination of fluconazole with the octyl derivative at the same concentration was the most effective among the tested conditions, resulting in a less dense biofilm structure and increased blue fluorescence, indicating a higher number of dead cells and a significant decrease in viability. For *C. gattii* biofilms (Fig. 4A1 through D2/2), a similar response pattern was observed.

### Characterization of biofilms (post-treatment)

**Fig 5:**
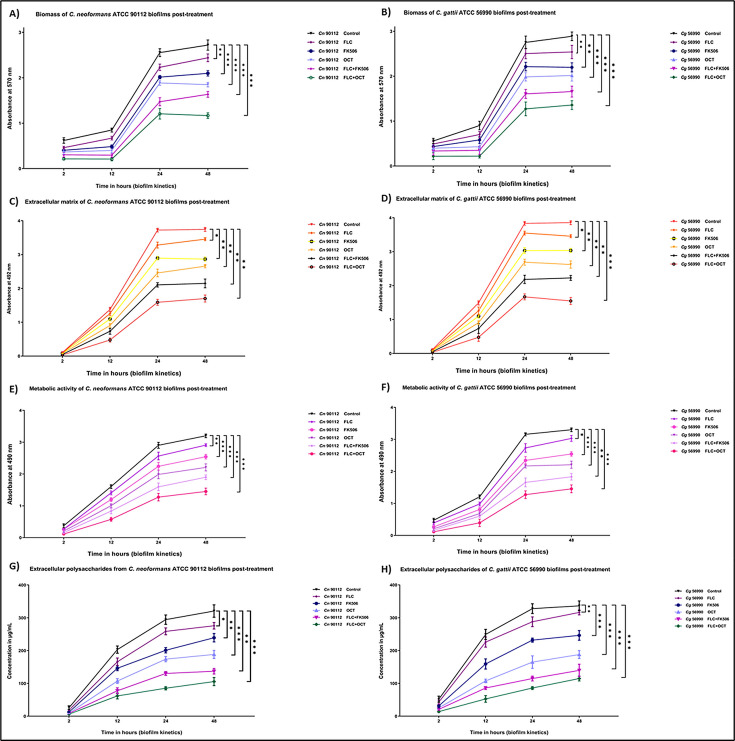
(A and B) Biomass levels after treatment in *C. neoformans* and *C. gattii* biofilms, respectively. Statistical comparisons indicated (* = significance). (C and D) Extracellular matrix levels. (E and F) Metabolic activity. (G and H) Extracellular polysaccharide levels. Control: untreated biofilm sample.

After determining the MICs, selected compounds were applied at the lowest MIC for each biofilm stage to compare efficacy (Fig. 5A through H, Table 4). Untreated biofilms of both species grew robustly over time. Fluconazole alone showed limited inhibition; FK506 was more effective; the octyl derivative was most effective as a monotherapy. Combined fluconazole + FK506 caused substantial reductions; fluconazole + octyl was consistently the most effective combination, producing the most pronounced reductions in all parameters.

### Quantification of target gene expression in biofilms (post-treatment)

**Fig 6:**
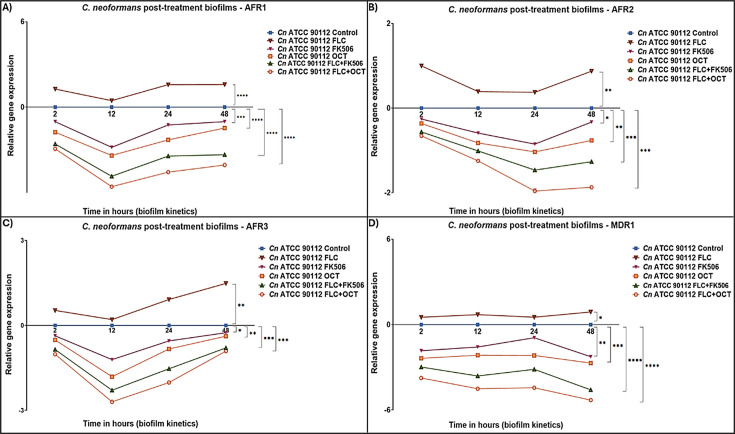
AFR1, AFR2, AFR3, and MDR1 efflux pump gene expression in the biofilm form of *C. neoformans* ATCC 90112 following treatment. (**A**) AFR1. (**B**) AFR2. (**C**) AFR3. (**D**) MDR1. Gene expression control: respective untreated biofilm samples. All comparisons were statistically significant (* = significance).

**Fig 7:**
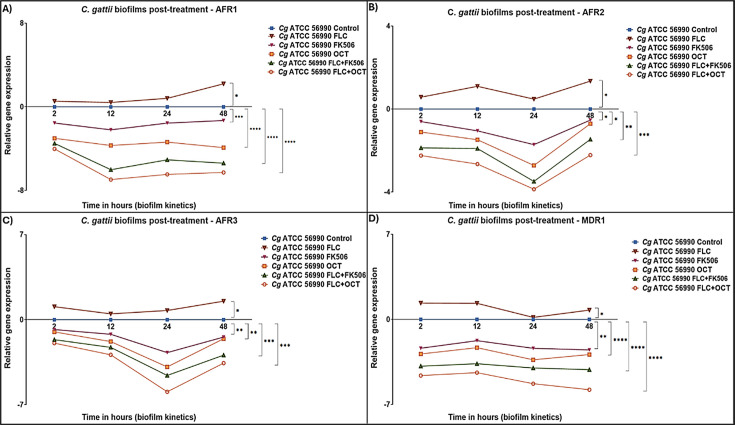
AFR1, AFR2, AFR3, and MDR1 efflux pump gene expression in the biofilm form of *C. gattii* ATCC 56990 following treatment. (**A**) AFR1. (**B**) AFR2. (**C**) AFR3. (**D**) MDR1. Gene expression control: respective untreated biofilm samples. All comparisons were statistically significant (* = significance).

The same concentrations were used for all tested compounds, corresponding to suboptimal doses (2 × lower than MIC) of the most effective treatments observed (FLC + OCT). In both species (Fig. 6 and 7), treatment with fluconazole alone significantly increased the expression of efflux pump genes. The combination of fluconazole with octyl derivative was particularly effective in reducing the expression of these pumps, indicating that octyl may potentiate the antifungal effect of fluconazole. The fluconazole + FK506 combination also reduced gene expression, but to a lesser extent than fluconazole + octyl. All treatments, except fluconazole alone, induced the downregulation of the efflux pump genes evaluated.

### Recovery and induction of persister cells

**Fig 8:**
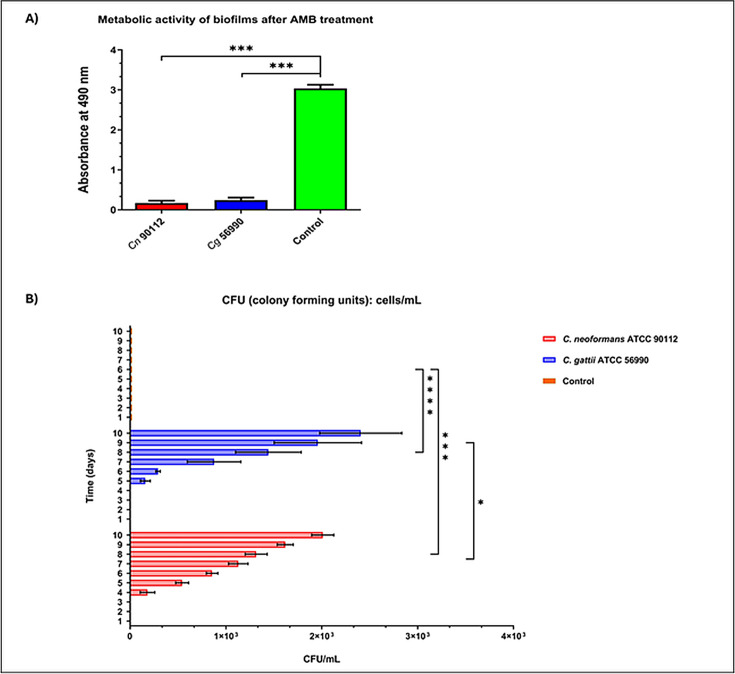
(**A**) Metabolic activity of *C. neoformans* ATCC 90112 and *C. gattii* ATCC 56990 biofilms treated with 100 µg/mL amphotericin B for 48 h. Growth control–Cn 90112: *P* < 0.0008***. Growth control–Cg 56990: *P* < 0.0006***. (**B**) Colony-forming units after biofilm treatment and resuspension on Sabouraud Dextrose agar. Medium control–Cn 90112: *P* < 0.0008***. Medium control–Cg 56990: *P* < 0.00009****. Cn 90112–Cg 56990: *P* < 0.0486*.

In graph A (Fig. 8), it can be observed that the metabolic activity of *C. neoformans* and *C. gattii* biofilms was significantly reduced compared to the growth control. The absorbance of *C. neoformans* and *C. gattii* biofilms was almost null, whereas the control showed an absorbance of approximately 3.0. Regarding graph B, it is noted that even after the biofilms were subjected to selective pressure, with extensive damage and drastic reduction in metabolic activity due to exposure to very high concentrations of AMB, growth still occurred, albeit more slowly and gradually, of the persister cells on Sabouraud Dextrose agar. Therefore, results indicate that amphotericin B treatment was highly effective in reducing the metabolic activity and cell viability of *C. neoformans* and *C. gattii* biofilms, exerting significant selective pressure and enabling the isolation of persister cells from the rest of the biofilm populations.

### Multiphoton microscopy of persister cells

**Fig 9:**
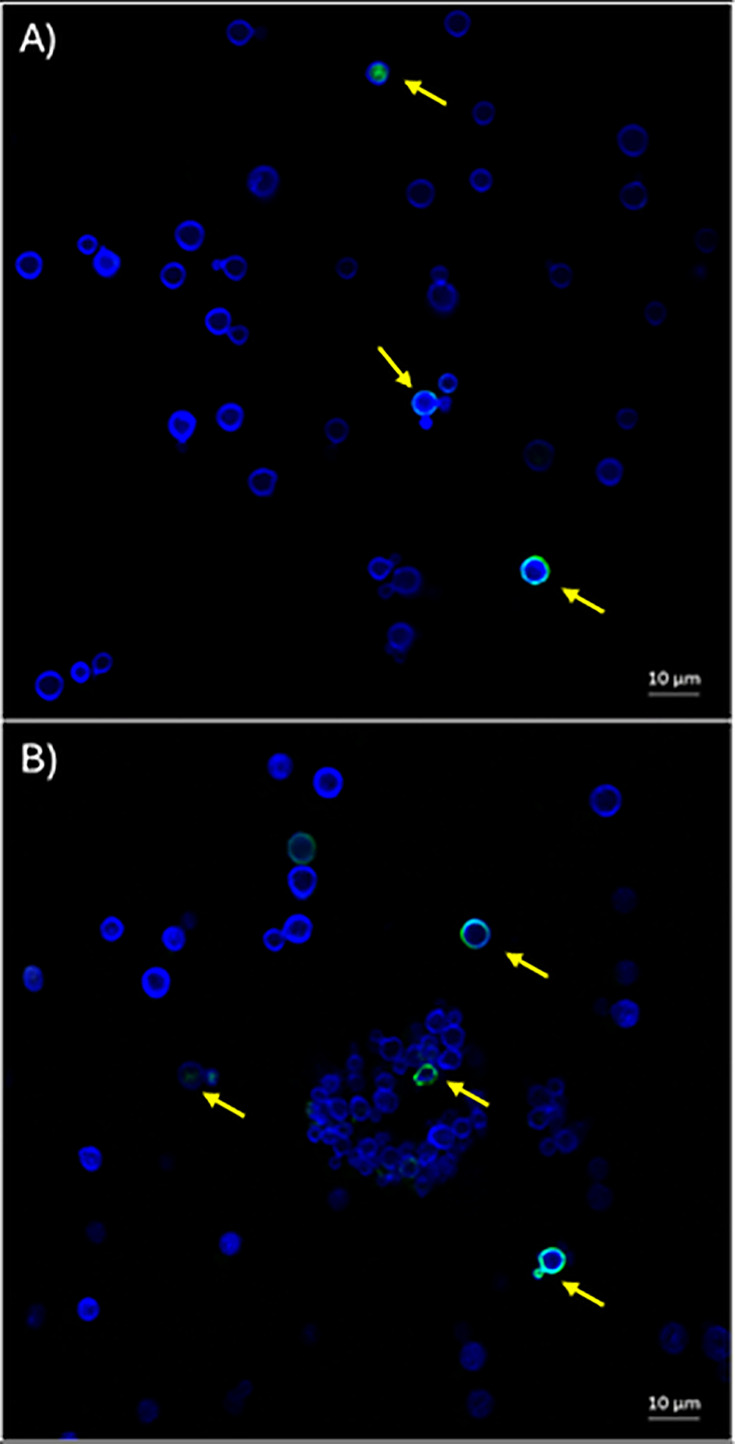
Multiphoton microscopy of *C. neoformans* ATCC 90112 and *C. gattii* ATCC 56990 biofilms. (**A and B**) Mature biofilm cells treated with 100 µg/mL amphotericin B for 48 h and stained with Calcofluor White (blue; fungal cell wall) and Diacetate Fluorescein (green; viable cells). Yellow targets: persister cells.

In both species (Fig. 9), persister cells were visualized with a greenish coloration transitioning between stronger and weaker/less intense shades. All other cells were dead and stained with Calcofluor White. Treatment with amphotericin B resulted in the survival of only 1%–2% of the cells present in mature biofilms according to the visualization shields.

### Determination of MIC and MFC of persister cells against selected compounds

The results indicate that the antifungal combinations fluconazole + FK506 and fluconazole + octyl are significantly more effective at inhibiting the growth of persister cells compared to the use of each antifungal alone (Tables 6 and 7). The fluconazole + octyl combination was the most effective, reducing the MIC to 64 μg/mL for *C. neoformans* and 128 μg/mL for *C. gattii*, although both species maintained a resistant profile. Fractional inhibitory concentration index (FICI) values below 1 indicate synergy between the antifungals. Here, the FICI values for both antifungal combinations were well below 1, demonstrating synergistic interactions. The fluconazole + octyl combination exhibited the strongest synergy (FICI = 0.156 for *C. neoformans* and 0.187 for *C. gattii*), confirming the MIC results (Table 8). The results also showed that the minimum fungicidal concentration (MFC) of amphotericin B was the same for both strains, suggesting that persister cells of *C. neoformans* and *C. gattii* have similar sensitivity to amphotericin B. However, they exhibited a high resistance profile, exceeding four times the induction dose (100 μg/mL) and more than one thousand times the fungicidal dose for planktonic cells (0.250 μg/mL for *C. neoformans* and 0.500 μg/mL for *C. gattii*).

**TABLE 6:** Minimum inhibitory concentration (MIC) to inhibit the growth of persister cells

Minimum inhibitory concentration (MIC)—persister cells					
Strain	Fluconazole	FK506	Octyl	Fluconazole + FK506	Fluconazole + octyl
ATCC *Cn* 90112	2,048 µg/mL	512 µg/mL	512 µg/mL	128 µg/mL	64 µg/mL
ATCC *Cg* 56990	2,048 µg/mL	1,024 µg/mL	1,024 µg/mL	256 µg/mL	128 µg/mL

**TABLE 7:** Minimum fungicidal concentration (MFC) of persister cells

Minimum fungicidal concentration (MFC)—planktonic cells	
Strain	Amphotericin B
ATCC *Cn* 90112	0.250 µg/mL
ATCC *Cg* 56990	0.500 µg/mL

Minimum fungicidal concentration (MFC)—persister cells	
ATCC *Cn* 90112	400 µg/mL
ATCC *Cg* 56990	400 µg/mL

**TABLE 8:** Fractional inhibitory concentration index (FICI)—persister cells

Fractional inhibitory concentration index (FICI)—persister cells		
Strain	Fluconazole + FK506	Fluconazole + octyl
ATCC *Cn* 90112	0.312	0.156
ATCC *Cg* 56990	0.375	0.187

### Quantification of target gene expression in persister cells

**Fig 10:**
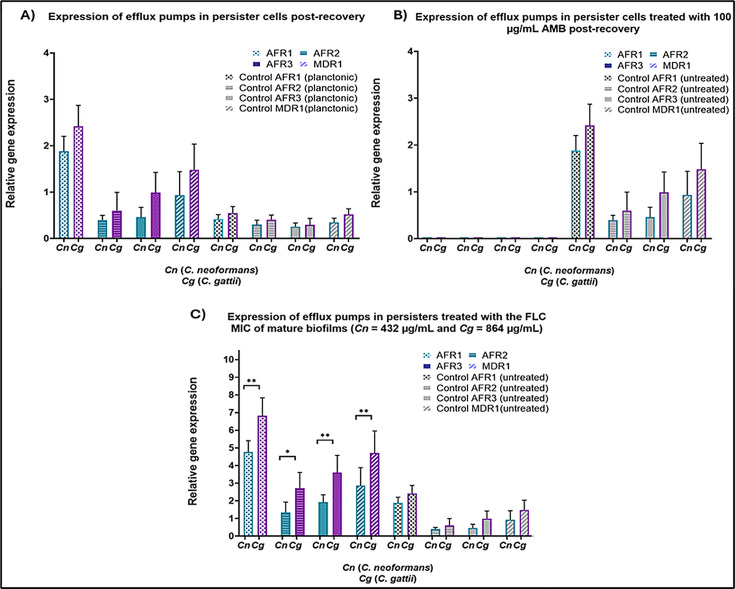
(**A**) Efflux pump expression in persister cells of *C. neoformans* ATCC 90112 and *C. gattii* ATCC 56990 post-recovery. Gene expression controls: planktonic samples. (**B**) Efflux pump expression in persister cells treated with 100 µg/mL amphotericin B after recovery. Gene expression controls: respective untreated persister samples. (**C**) Efflux pump expression in persister cells following post-recovery treatment with the MIC of fluconazole from mature biofilms (Cn = 432 µg/mL; Cg = 864 µg/mL). AFR1 Cn–Cg: *P* < 0.0015**; AFR2: *P* < 0.020*; AFR3: *P* < 0.0015**; MDR1: *P* < 0.0029**. Gene expression controls: respective untreated persister samples.

After the recovery period (10 days), persister cells of *C. neoformans* and *C. gattii* exhibited minimal but relevant overexpression of efflux pump genes (Fig. 10A). *AFR1* was the most highly expressed gene in both species, while AFR2 showed the lowest expression. *C. gattii* maintained higher expression levels than *C. neoformans*. High-dose AMB (100 μg/mL) caused a drastic reduction, effectively abolishing the expression of all evaluated efflux pump genes (Fig. 10B). Persister cells treated with the MIC of fluconazole from mature biofilms exhibited the overexpression of all four efflux pump genes in both species (Fig. 10C), underscoring the insufficiency of this antifungal and confirming that *C. gattii* showed greater relative induction of *AFR1* compared to *C. neoformans*.

## DISCUSSION

**Fig 11:**
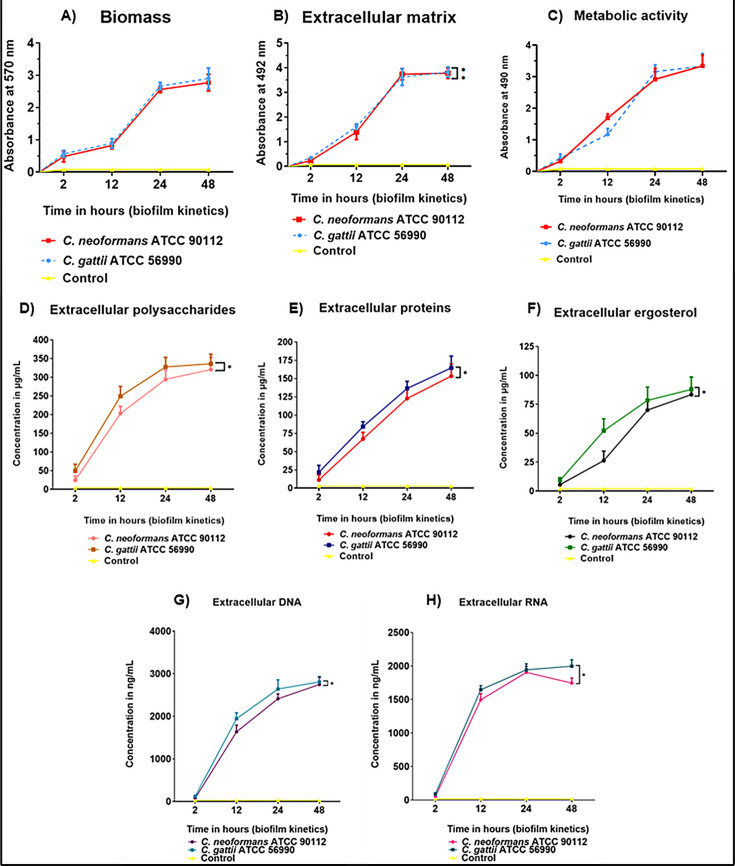
(**A**) Biomass levels during biofilm formation kinetics of *C. neoformans* ATCC 90112 and *C. gattii* ATCC 56990. (**B**) Extracellular matrix levels (*P* < 0.0098**). (**C**) Mitochondrial metabolic activity. (**D**) Extracellular polysaccharide quantification (*P* < 0.0199*). (**E**) Extracellular protein quantification (*P* < 0.0193*). (**F**) Extracellular ergosterol quantification (*P* < 0.0259*). (**G**) Extracellular DNA quantification (*P* < 0.0437*). (**H**) Extracellular RNA quantification (*P* < 0.0337*). Controls: growth medium without fungal inoculum.

Fungal biofilms represent the main form of microbial growth that significantly contributes to treatment resistance and the persistence of infections. The ability to form biofilms provides fungi with adaptive advantages, such as adherence to favorable growth sites, protection against desiccation, predation, and resistance to biocides. The capacity of *Cryptococcus* to establish biofilms on both abiotic surfaces (e.g., medical devices) and biotic substrates has direct implications for the pathogenesis of infections caused by these microorganisms (29). Yeast cells may exploit this adaptive condition to gain access to the bloodstream and subsequently invade internal organs. This scenario is particularly concerning, as disseminated fungal infections are associated with high mortality rates (2, 30). The present study demonstrated that both *C. neoformans* and *C. gattii* strains exhibited a similar growth pattern and adhered consistently to the abiotic surface tested (polystyrene), forming dense and well-structured biofilms (Fig. 1, 2 and 11A through C), encompassing all stages in a coordinated manner (pre-adhesion, initial adhesion/microcolony formation, intermediate adhesion, and maturation). These findings reinforce the observations of Martinez and Casadevall (29).

The biofilm ECM is a complex protective network of proteins, polysaccharides, lipids, and nucleic acids (31). Cryptococcal polysaccharides act as a scaffold for structural support, protection, and adhesion (14, 32). Proteins participate in adhesion, stress defense, metabolic adjustments, and resistance (20). Ergosterol is essential for membrane integrity, affecting membrane ATPase activity; defects in its biosynthesis increase sensitivity to azoles and oxidative stress (33, 34). eDNA strengthens biofilm antifungal resistance in *C. albicans* and *A. fumigatus*, while eRNA confers mechanical stability (35–37). The ECM distinguishes biofilms from planktonic cells and facilitates resistance through efflux pump induction and protective structuring (38, 39).

According to the treatment results obtained in the present study, biofilms of *C. neoformans* and *C. gattii* were significantly less susceptible than planktonic cells to all compounds tested (Tables 2 and 4). These findings are consistent with other studies reporting that the biofilm phenotype confers resistance to antifungal therapy (8, 39). Biofilm formation is the most common mode of microbial growth in nature, representing approximately 65% of all clinical infections. Due to the resistance levels associated with this phenotype, biofilms pose a major obstacle in clinical practice (40). Planktonic cells generally rely on irreversible genetic modifications to sustain a resistant phenotype, whereas biofilms can persist through their physical presence and high cell density, which provides an intrinsically resistant phenotype, independent of defined genetic alterations. Furthermore, biofilms can be up to 1,000 times more resistant to antifungal agents than planktonic cells, although the mechanisms underlying this strong antagonism to antifungal therapy are not yet fully understood (38, 41). As observed in the susceptibility assays of early-stage biofilms (2 h and 12 h) in this study, although resistance is stronger and more robust in later phases of development, resistance to antifungal compounds can be detected just a few hours after surface adhesion even though MIC values are lower than those observed in mature biofilms (14, 42). Moreover, some studies suggest that the expression of multidrug resistance efflux pumps is higher in young biofilms and has been associated with increased resistance to azoles, although this mechanism does not appear to be exclusive or predominant in mature biofilm resistance (27, 38, 43, 44). An important detail, for example, is that the transcriptional profile of efflux pumps observed in *C. gattii* ATCC 24065 biofilms in studies such as that of Tavares et al. (44) was similar to that described for the reference strain R265 under infection conditions, suggesting that the pattern may be conserved. Our results (Fig. 11) can support this premise using ATCC’s, showing that efflux pump genes (*AFR1*, *AFR2*, *AFR3,* and *MDR1*—particularly *AFR1* and *MDR1*) display higher transcript expression in young biofilms (between 12 h and 24 h) compared to mature biofilms (48 h), even though mature biofilms (48 h) exhibited equal or higher MIC values than young biofilms.

**Fig 12:**
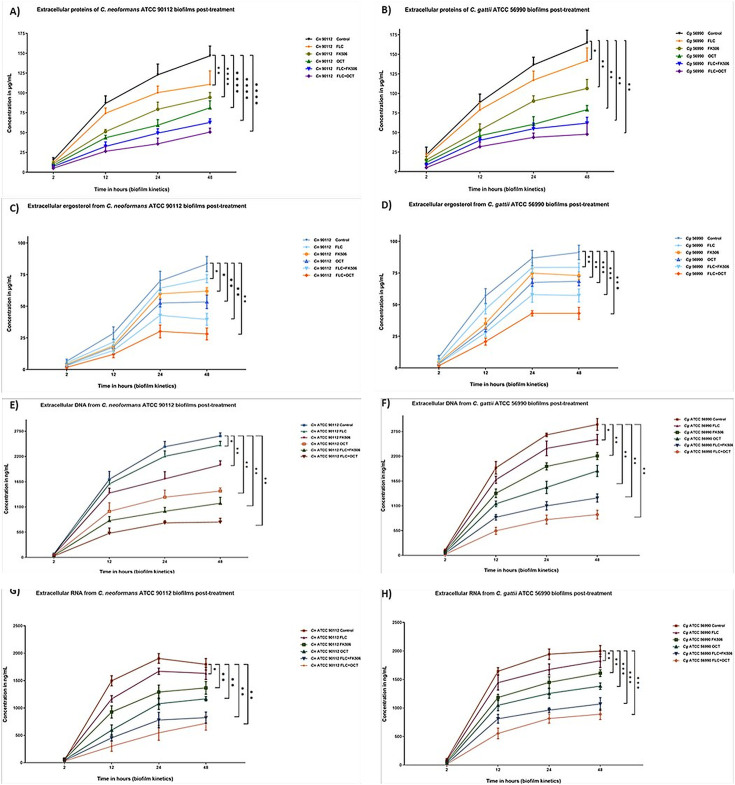
(**A and B**) Extracellular protein levels after treatment in *C. neoformans* and *C. gattii* biofilms. (**C and D**) Extracellular ergosterol levels. (**E and F**) Extracellular DNA levels. (**G and H**) Extracellular RNA levels. All comparisons were statistically significant (* = significance). Control: untreated biofilm sample.

In the present study, treatment results revealed that fluconazole, when used alone, was the compound with the highest MIC values, both for the planktonic form and especially for the biofilm form (where MIC values indicated resistance in both strains and species tested—Table 4). Previous studies have shown that resistance to azoles (such as fluconazole, itraconazole, and voriconazole) in *C. neoformans* can be associated with alterations in the target enzyme lanosterol 14α-demethylase, encoded by the *ERG11* gene (45–47). Resistance can also be related to transport changes caused by efflux pumps, such as Afr1 (typically composed of two transmembrane domains and two cytoplasmic domains) (7, 48, 49). In *Cryptococcus*, chromosome 1 harbors both the genes involved in ergosterol biosynthesis and the major efflux pump genes for azoles. Studies have shown that duplication of this chromosome can lead to heteroresistance to azoles and that both gene sets play a crucial role in this duplication, consequently driving phenotypic manifestations such as the overexpression of efflux pumps (47, 48, 50). ABC and MFS efflux pump genes are overexpressed in *C. gattii* biofilms according to mapped transcriptional profiles (44). Other studies also demonstrated that *Cryptococcus* biofilms can exhibit marked resistance to fluconazole, reporting MIC values well above 32 or 64 µg/mL (14, 51). In this context, although studies such as those by Stone et al. (47), Basso et al. (48), and Chang et al. (49) were conducted with clinical isolates, our results revealed a similar scenario, where ABC transporter genes (*AFR1, AFR2, AFR3*) and the MFS transporter gene (*MDR1*) were overexpressed (Fig. 3) in fluconazole-resistant natural biofilms in *C. neoformans* and, more prominently, in *C. gattii* (Table 4), reinforcing the translational relevance of the efflux pump expression patterns observed in the present study with ATCC reference strains. Although *AFR1* and *MDR1* are the genes most directly associated with fluconazole resistance and *AFR2* acts synergistically when co-overexpressed with *AFR1* and *MDR1* (49), recent evidence indicates that *AFR3* also contributes to resistance against this azole, even if less specifically. Inactivation of this transporter can result in increased susceptibility, reduced GXM, and decreased ergosterol content (52, 53). Comparing Fig. 6, 7, 11F and 12D, we can suggest that *AFR3* may be important for the maintenance of ergosterol content.

Although FK506 displayed a lower MIC than fluconazole alone, combined treatments proved more promising. Fluconazole + FK506 drastically reduced MIC values in both planktonic cells and biofilms (Tables 2 and 4), with FICI indices demonstrating significant synergy (Tables 3 and 5). Biofilm characterization showed that biomass, metabolic activity, and ECM components (polysaccharides, proteins, ergosterol, eDNA, and eRNA) were significantly affected (Fig. 5 and 12), as were efflux pump genes *AFR1, AFR2, AFR3*, and *MDR1* (Fig. 6 and 7). Calcineurin inhibitors suppress the fungal stress response, reducing MFS efflux pump expression and increasing intracellular FLC accumulation; our MDR1 expression results corroborate these findings (54, 55, 56, 57). Fluconazole + FK506 synergism in *C. neoformans* is driven by the inhibition of multidrug resistance pumps, with Afr1, Afr2, Afr3, and Mdr1 transmembrane domains showing affinity for FK506 substrates (58, 59). This synergism was clinically validated: Rossato et al. (60) demonstrated synergistic effects in 70% of *C. neoformans* isolates (60), and Reichert-Lima et al. (26) identified relevant synergistic interactions among 80 Brazilian clinical isolates (26).

Similarly, although the protocatechuic acid derivative octyl (octyl protocatechuate) exhibited a lower and more effective MIC than fluconazole, the results of the combined treatments in both planktonic cells and biofilms proved to be more promising. The combination of fluconazole + octyl significantly reduced MIC values when compared to the compounds used individually (Tables 2 and 4). The FICI indices demonstrated a significant effect among the combinations tested (Tables 3 and 5). Biofilm characterization after treatment clearly revealed the phenotypes observed. In this characterization, all compounds were applied at the same concentration, corresponding to the lowest MIC value determined for each phase of biofilm formation (see Table 4) in order to allow direct comparison of antifungal and antibiofilm efficacy. The expression of all target genes (*AFR1, AFR2, AFR3*, and *MDR1—*Fig. 6 and 7) was significantly reduced. Moreover, both biomass and metabolic activity, as well as the extracellular matrix and its main components (polysaccharides, proteins, ergosterol, eDNA, and eRNA), were affected by the synergism between fluconazole + octyl. As demonstrated by Soares (12), Medina-Alarcón (61), and collaborators, octyl protocatechuate acts synergistically with antifungals such as fluconazole, enhancing damage to the cell membrane and potentiating antifungal activity against *Trichophyton* and *P. brasiliensis* and *P. lutzii*, respectively, while showing very low toxicity in pulmonary fibroblast lineage MRC5 (98% viability). Another study reported that protocatechuic acids can inhibit biofilm formation and enhance antifungal efficacy against *Candida albicans* (62). Treatment with nonyl protocatechuate in *T. rubrum* and *T. mentagrophytes* caused severe biofilm damage: cell wall collapse and leakage of cytoplasmic content. The antimicrobial activity of phenolic acid derivatives increases with the number of alkyl side chains (12, 63). The hydrophilic portion of phenolic compounds can bind to the hydrophilic region of the cell membrane, allowing penetration and disruption of lipid bilayers (64). These results demonstrate that this compound primarily targets the cell membrane by an amphiphilic nature, disrupting the plasma membrane and altering its fluidity and integrity, mainly through interference with ergosterol biosynthesis and downregulation of the *ERG11* gene (65). Similarly, another phenolic compound, gallic acid, also exhibited antifungal activity when combined with fluconazole, reducing MIC values. This acid was able to affect the expression of key efflux pump genes in *Cryptococcus gattii*, namely, *AFR1* and *MDR1* (66).

Phenotypically, treatments with FK506 and octyl—alone and combined with fluconazole—highlighted a crucial role of efflux pumps in biofilms. FK506 reduces and inhibits these pumps (58, 67); similar effects occur with protocatechuic acid derivatives (66). These inhibitory effects were observed across all biofilm formation, also reducing the components of the ECM components—consistent with the role of ABC and MFS transporters in exporting quorum sensing mediators and polymeric substances (33, 68, 69). Their inhibition by FK506 and octyl enhanced FLC efficacy through increased intracellular ergosterol availability, disrupting membrane homeostasis and reducing biofilm density (Fig. 5 and 12, Table 4).

Persister cells are consistently identified in a “dormant” state, which is directly associated with reduced metabolic activity. As shown in (Fig. 10A), the identified cells exhibited low metabolic activity, possibly corroborating the dormancy characteristics of persister cells observed in biofilms of other fungi (70). The main glucose-utilization pathways, such as glycolysis, and pentose phosphate pathway, are negatively regulated in persister cells (70, 71). Studies indicate that the downregulation of glycolytic proteins in persister cells results in impaired energy storage and may also increase the rigidity of the plasma membrane, thereby hindering the action of amphotericin B. Glucose deprivation induces a dormant state, as observed in these cells. It is important to note that while similar effects occur in glucose-deprived planktonic cultures, no persister cells are observed under those conditions. In the absence of glucose, fungal cells are forced to utilize alternative carbon sources (oxaloacetate, phosphoenolpyruvate, and fructose-1,6-bisphosphate), which yield trehalose and glycogen as products. The proteomic signature of persister cells from *Candida albicans* and *Saccharomyces cerevisiae* suggests that these cells can survive using such alternative carbon sources (9, 70).

Biofilms treated with 100 μg/mL AMB for 48 h confirmed selective pressure, leaving only persister cells assessed by CFU and multiphoton microscopy (Fig. 8B and 9) (27). Slow but consistent colony growth was observed for up to 10 days. MIC tests (Table 6) and MFC tests (Table 7) demonstrate extreme tolerance to fluconazole (MIC 2,048 μg/mL) and AMB (MFC 400 μg/mL—>1,000-fold above planktonic fungicidal doses). Tolerance implies survival after transient exposure to fungicidal concentrations; persisters represent a primary case of this phenomenon (72). A biphasic killing pattern is observed: most cells are killed, but a small proportion survives and can reproduce the pattern upon re-exposure (38, 73, 74). The development of AMB resistance is rare, confirming that surviving colonies represent genuine persister cells (75, 76).

Our results showed that the main efflux pumps of persister cells from *C. neoformans* and *C. gattii* are virtually not expressed during amphotericin B treatment (Fig. 10B). This suggests that ABC efflux pumps may not be crucial for the survival of these cells during exposure to high doses of amphotericin B. One indication supporting this hypothesis is that energy availability (ATP) is stored as a reserve in these cells during stress, allowing them to resume proliferation under favorable conditions later (9, 27). This premise aligns with our data, which showed the reactivation of these pumps once favorable conditions were restored (Fig. 10A).

Treatment with FK506 and the octyl derivative alone, as well as their combination with fluconazole, reduced the MICs of persister cells. This demonstrates that, in conjunction with the gene expression analysis after recovery (Fig. 10A) and during subsequent exposure to fluconazole (Fig. 10C), these cells reactivated the expression of ATP-dependent efflux pumps (ABC). Literature and biofilm data indicate that these transporters, in addition to MFS transporters, are targets of FK506 and phenolic compounds. Finally, the efficacy of the fluconazole + FK506 and fluconazole + octyl combinations was notable (Table 8), significantly reducing these MICs and suggesting that such combinations could represent promising alternatives in the complex context of cryptococcosis therapy.

From a specific clinical standpoint, the results of this study may lead to significant implications for the management of recurrent and persistent cryptococcal infections (77, 78). Persister cells, by surviving high concentrations of antifungals in a dormant state and subsequently reactivating the expression of the efflux pump, represent a plausible biological basis for treatment failure and infection recurrence—particularly in immunocompromised patients undergoing prolonged maintenance antifungal therapy with fluconazole. The ability of persistent biofilm-derived cells to resist amphotericin B concentrations far exceeding the standard fungicidal dose for planktonic forms underscores why conventional therapeutic regimens may prove insufficient in the context of device-associated or tissue-disseminated cryptococcal infections. In this scenario, the synergistic combinations explored here—fluconazole + FK506 and fluconazole + octyl derivative—offer a double advantage: greater antifungal efficacy and potentially a reduction in the total drug burden (46, 47, 78–81). By reducing the effective inhibitory concentrations required, these combinations could mitigate the risks of nephrotoxicity and hepatotoxicity associated with high doses of amphotericin B or prolonged azole regimens, respectively (54, 55, 61, 82). These observations encourage further preclinical and clinical investigations of combination strategies targeting both efflux-mediated resistance and biofilm persistence as complementary approaches to reduce cryptococcosis treatment failure (8, 9, 67).

### Conclusions

*C. neoformans* and *C. gattii* biofilms exhibit structural complexity and heterogeneity, with a matrix rich in biomolecules essential for their development. Efflux pumps play a crucial role in biofilm formation and maintenance, aiding in the transport of extracellular molecules and contributing to antifungal resistance. Treatments with fluconazole combined with FK506, and particularly with the octyl derivative, have demonstrated greater efficacy in disrupting biofilm structure and in the formation and expression of the studied efflux pump genes. The synergistic combinations identified in this study offer a promising pathway to reduce total antifungal exposure—and, therefore, the nephrotoxic and hepatotoxic burden associated with conventional regimens—while maintaining or improving therapeutic efficacy. Persister cells of these species can survive high concentrations of antifungals, alternating between dormant and active states. During exposure to amphotericin B, efflux pumps were not essential for their survival, although their expression was subsequently reactivated. Combinations of fluconazole + FK506 and fluconazole + octyl (especially) were more effective against these cells. Clinically, the persistence of cells embedded in biofilms, capable of reactivating efflux pump expression after exposure to antifungals, may lead to a possible mechanistic explanation for recurrent cryptococcal infections (particularly in immunocompromised patients) on prolonged maintenance therapy. These findings elucidate resistance mechanisms in biofilms, point to the presence of persister cells, and suggest potential new therapeutic strategies for cryptococcosis. It is important to mention, however, that this study was conducted with ATCC reference strains (*C. neoformans* ATCC 90112 and *C. gattii* ATCC 56990). Clinical isolates may exhibit distinct phenotypic and genotypic profiles due to prior exposure to antifungal agents, host immune pressures, and environmental stressors, which can lead to divergent biofilm formation and persister cell capacities, as well as distinct efflux pump expression patterns. Future investigations comparing ATCC reference strains with clinical isolates—including those recovered from patients with recurrent or refractory cryptococcosis—will be crucial to validate and expand the biological and clinical relevance of these findings.

## Data Availability

The data supporting the findings of this study are available from the corresponding author upon reasonable request. No novel sequences or data sets were deposited in public repositories.

## References

[1] World Health Organization. 2022. Fungal priority pathogens list to guide research, development and public health action. Geneva, Switzerland WHO

[2] Denning DW. 2024. Global incidence and mortality of severe fungal disease. Lancet Infect Dis 24:e428–e438. doi:10.1016/S1473-3099(23)00692-8 38224705

[3] Firacative C, Meyer W, Castañeda E. 2021. *Cryptococcus neoformans* and *Cryptococcus gattii* species complexes in latin america: a map of molecular types, genotypic diversity, and antifungal susceptibility as reported by the Latin American cryptococcal study group. JoF 7:282. doi:10.3390/jof7040282 33918572 PMC8069395

[4] Firacative C, Trilles L, Meyer W. 2021. Recent advances in *Cryptococcus* and cryptococcosis. Microorganisms 10:13. doi:10.3390/microorganisms10010013 35056462 PMC8779235

[5] White TC, Marr KA, Bowden RA. 1998. Clinical, cellular, and molecular factors that contribute to antifungal drug resistance. Clin Microbiol Rev 11:382–402. doi:10.1128/CMR.11.2.382 9564569 PMC106838

[6] Chang A. 2020. Mitochondrial morphology, oxidative stress resistance, and pathogenesis in *Cryptococcus neoformans* PhD dissertation, Washington University in St. Louis, MO

[7] Sanglard D, Coste A, Ferrari S. 2009. Antifungal drug resistance mechanisms in fungal pathogens from the perspective of transcriptional gene regulation. FEMS Yeast Res 9:1029–1050. doi:10.1111/j.1567-1364.2009.00578.x 19799636

[8] Lee Y, Robbins N, Cowen LE. 2023. Molecular mechanisms governing antifungal drug resistance. NPJ Antimicrob Resist 1:5. doi:10.1038/s44259-023-00007-2 38686214 PMC11057204

[9] Wuyts J, Van Dijck P, Holtappels M. 2018. Fungal persister cells: the basis for recalcitrant infections? PLoS Pathog 14:e1007301. doi:10.1371/journal.ppat.1007301 30335865 PMC6193731

[10] Soares LA. 2011. Estudo da atividade antidermatofítica de protocatecuatos contra *T. rubrum* e *T. interdigitale* Master’s thesis, Universidade Estadual Paulista, Araraquara, Brazil

[11] Daglia M. 2012. Polyphenols as antimicrobial agents. Curr Opin Biotechnol 23:174–181. doi:10.1016/j.copbio.2011.08.007 21925860

[12] Soares LA, Gullo FP, Sardi J de C, Pitangui N de S, Costa-Orlandi CB, Sangalli-Leite F, Scorzoni L, Regasini LO, Petrônio MS, Souza PF, et al.. 2014. Anti-trichophyton activity of protocatechuates and their synergism with fluconazole. Evid Based Complement Alternat Med 2014:957860. doi:10.1155/2014/957860 25136374 PMC4086519

[13] Jaiswal N, Kumar A. 2024. Modulators of Candida albicans Membrane Drug Transporters: A Lucrative Portfolio for the Development of Effective Antifungals. Mol Biotechnol 66:960–974. doi:10.1007/s12033-023-01017-1 38206530

[14] Martinez LR, Casadevall A. 2006. Susceptibility of *Cryptococcus neoformans* biofilms to antifungal agents *in vitro*. Antimicrob Agents Chemother 50:1021–1033. doi:10.1128/AAC.50.3.1021-1033.2006 16495265 PMC1426450

[15] Lima Gualque MW Vaso CO Santos KS Galeane MC, Gomes PC, Palma MS, Mendes Giannini MJS, Fusco Almeida AM. 2024. Peptides from *Galleria mellonella* against *Cryptococcus* spp.: toxicity in three-dimensional cell cultures and *G. mellonella*. Future Microbiol 20:11–21. doi:10.1080/17460913.2024.2421632 39552598 PMC11731228

[16] Martins M, Uppuluri P, Thomas DP, Cleary IA, Henriques M, Lopez-Ribot JL, Oliveira R. 2010. Presence of extracellular DNA in the *Candida albicans* biofilm matrix and its contribution to biofilms. Mycopathologia 169:323–331. doi:10.1007/s11046-009-9264-y 20012895 PMC3973130

[17] Marcos-Zambrano LJ, Escribano P, Bouza E, Guinea J. 2014. Production of biofilm by *Candida* and non-*Candida* spp. isolates causing fungemia: comparison of biomass production and metabolic activity and development of cut-off points. Int J Med Microbiol 304:1192–1198. doi:10.1016/j.ijmm.2014.08.012 25224357

[18] Costa-Orlandi CB, Sardi JCO, Santos CT, Fusco-Almeida AM, Mendes-Giannini MJS. 2014. *In vitro* characterization of *Trichophyton rubrum* and *T. mentagrophytes* biofilms. Biofouling 30:719–727. doi:10.1080/08927014.2014.919282 24856309

[19] DuBois Michel, Gilles KA, Hamilton JK, Rebers PA, Smith Fred. 1956. Colorimetric method for determination of sugars and related substances. Anal Chem 28:350–356. doi:10.1021/ac60111a017

[20] Santi L, Beys-da-Silva WO, Berger M, Calzolari D, Guimarães JA, Moresco JJ, Yates JR III. 2014. Proteomic profile of *Cryptococcus neoformans* biofilm reveals changes in metabolic processes. J Proteome Res 13:1545–1559. doi:10.1021/pr401075f 24467693 PMC3993910

[21] Bradford MM. 1976. A rapid and sensitive method for the quantitation of microgram quantities of protein utilizing the principle of protein-dye binding. Anal Biochem 72:248–254. doi:10.1016/0003-2697(76)90527-3 942051

[22] Pinheiro CG, Bianchini NH, Pavlack AS, Muniz MFB, Barboza VDS, Pavanato MA, Gouveia FN, Heinzmann BM. 2020. A method for evaluating ergosterol content in wood-decay fungi. Rev Árvore 44:e4432. doi:10.1590/1806-908820200000010

[23] Oliveira LT, Marcos CM, Cabral AKLF, Medina-Alarcón KP, Pires RH, Fusco-Almeida AM, Mendes-Giannini MJS. 2024. *Paracoccidioides* spp.: the structural characterization of extracellular matrix, expression of glucan synthesis and associated genes and adhesins during biofilm formation. Front Microbiol 15:1354140. doi:10.3389/fmicb.2024.1354140 38516014 PMC10955377

[24] Benaducci T, Sardi J de CO, Lourencetti NMS, Scorzoni L, Gullo FP, Rossi SA, Derissi JB, de Azevedo Prata MC, Fusco-Almeida AM, Mendes-Giannini MJS. 2016. Virulence of *Cryptococcus* sp. biofilms *in vitro* and *in vivo* using *Galleria mellonella* as an alternative model. Front Microbiol 7:290. doi:10.3389/fmicb.2016.00290 27014214 PMC4783715

[25] Clinical and Laboratory Standards Institute. 2008. Reference method for broth dilution antifungal susceptibility testing of yeasts, 3rd ed. Approved standard M27-A3. Wayne, PA CLSI

[26] Reichert-Lima F, Busso-Lopes AF, Lyra L, Peron IH, Taguchi H, Mikami Y, Kamei K, Moretti ML, Schreiber AZ. 2016. Evaluation of antifungal combination against *Cryptococcus* spp. Mycoses 59:585–593. doi:10.1111/myc.12510 27135278

[27] LaFleur MD, Kumamoto CA, Lewis K. 2006. *Candida albicans* biofilms produce antifungal-tolerant persister cells. Antimicrob Agents Chemother 50:3839–3846. doi:10.1128/AAC.00684-06 16923951 PMC1635216

[28] de Aguiar Cordeiro R, Serpa R, Flávia Uchoa Alexandre C, de Farias Marques FJ, Vladia Silva de Melo C, da Silva Franco J, José de Jesus Evangelista A, Pires de Camargo Z, Samia Nogueira Brilhante R, Fabio Gadelha Rocha M, et al.. 2015. *Trichosporon inkin* biofilms produce extracellular proteases and exhibit resistance to antifungals. J Med Microbiol 64:1277–1286. doi:10.1099/jmm.0.000159 26310576

[29] Martinez LR, Casadevall A. 2007. *Cryptococcus neoformans* biofilm formation depends on surface support and carbon source and reduces fungal cell susceptibility to heat, cold, and UV light. Appl Environ Microbiol 73:4592–4601. doi:10.1128/AEM.02506-06 17513597 PMC1932807

[30] Verstrepen KJ, Klis FM. 2006. Flocculation, adhesion and biofilm formation in yeasts. Mol Microbiol 60:5–15. doi:10.1111/j.1365-2958.2006.05072.x 16556216

[31] Mitchell KF, Zarnowski R, Andes DR. 2016. The extracellular matrix of fungal biofilms, p 21–35. *In* Advances in experimental medicine and biology. Springer International Publishing, Cham, Switzerland. 10.1007/5584_2016_6 27271680

[32] Martinez LR, Casadevall A. 2005. Specific antibody can prevent fungal biofilm formation and this effect correlates with protective efficacy. Infect Immun 73:6350–6362. doi:10.1128/IAI.73.10.6350-6362.2005 16177306 PMC1230912

[33] Zhang Y-Q, Gamarra S, Garcia-Effron G, Park S, Perlin DS, Rao R. 2010. Requirement for ergosterol in V-ATPase function underlies antifungal activity of azole drugs. PLoS Pathog 6:e1000939. doi:10.1371/journal.ppat.1000939 20532216 PMC2880581

[34] Kodedová M, Sychrová H. 2015. Changes in the sterol composition of the plasma membrane affect membrane potential, salt tolerance and the activity of multidrug resistance pumps in *Saccharomyces cerevisiae*. PLoS One 10:e0139306. doi:10.1371/journal.pone.0139306 26418026 PMC4587746

[35] Rajendran R, Williams C, Lappin DF, Millington O, Martins M, Ramage G. 2013. Extracellular DNA release acts as an antifungal resistance mechanism in mature *Aspergillus fumigatus* biofilms. Eukaryot Cell 12:420–429. doi:10.1128/EC.00287-12 23314962 PMC3629765

[36] Rajendran R, Sherry L, Lappin DF, Nile CJ, Smith K, Williams C, Munro CA, Ramage G. 2014. Extracellular DNA release confers heterogeneity in *Candida albicans* biofilm formation. BMC Microbiol 14:232. doi:10.1186/s12866-014-0303-6 25476750 PMC4262977

[37] Mugunthan S, Wong LL, Winnerdy FR, Summers S, Bin Ismail MH, Foo YH, Jaggi TK, Meldrum OW, Tiew PY, Chotirmall SH, et al.. 2023. RNA is a key component of extracellular DNA networks in *Pseudomonas aeruginosa* biofilms. Nat Commun 14:7772. doi:10.1038/s41467-023-43533-3 38012164 PMC10682433

[38] Ramage G, Rajendran R, Sherry L, Williams C. 2012. Fungal biofilm resistance. Int J Microbiol 2012:528521. doi:10.1155/2012/528521 22518145 PMC3299327

[39] Cowen LE, Sanglard D, Howard SJ, Rogers PD, Perlin DS. 2015. Mechanisms of antifungal drug resistance. Cold Spring Harb Perspect Med 5:a019752. doi:10.1101/cshperspect.a019752 PMC4484955 25384768

[40] Ramage G, Williams C. 2013. The clinical importance of fungal biofilms. Adv Appl Microbiol 84:27–83. doi:10.1016/B978-0-12-407673-0.00002-3 23763758

[41] Martinez LR, Fries BC. 2010. Fungal biofilms: relevance in the setting of human disease. Curr Fungal Infect Rep 4:266–275. doi:10.1007/s12281-010-0035-5 21660222 PMC3109917

[42] Finkel JS, Mitchell AP. 2011. Genetic control of *Candida albicans* biofilm development. Nat Rev Microbiol 9:109–118. doi:10.1038/nrmicro2475 21189476 PMC3891587

[43] Taff HT, Mitchell KF, Edward JA, Andes DR. 2013. Mechanisms of *Candida biofilm* drug resistance. Future Microbiol 8:1325–1337. doi:10.2217/fmb.13.101 24059922 PMC3859465

[44] Tavares ER, Gionco B, Morguette AEB, Andriani GM, Morey AT, do Carmo AO, de Pádua Pereira U, Andrade G, de Oliveira AG, Pinge-Filho P, et al.. 2019. Phenotypic characteristics and transcriptome profile of *Cryptococcus gattii* biofilm. Sci Rep 9:6438. doi:10.1038/s41598-019-42896-2 31015652 PMC6478838

[45] Rodero L, Mellado E, Rodriguez AC, Salve A, Guelfand L, Cahn P, Cuenca-Estrella M, Davel G, Rodriguez-Tudela JL. 2003. G484S amino acid substitution in lanosterol 14-alpha demethylase (ERG11) is related to fluconazole resistance in a recurrent *Cryptococcus neoformans* clinical isolate. Antimicrob Agents Chemother 47:3653–3656. doi:10.1128/AAC.47.11.3653-3656.2003 14576140 PMC253790

[46] Almeida AMF, Matsumoto MT, Baeza LC, de Oliveira E Silva RB, Kleiner AAP, Melhem M de S, Mendes Giannini MJS. 2007. Molecular typing and antifungal susceptibility of clinical sequential isolates of *Cryptococcus neoformans* from Sao Paulo State, Brazil. FEMS Yeast Res 7:152–164. doi:10.1111/j.1567-1364.2006.00128.x 17311593

[47] Stone NRH, Rhodes J, Fisher MC, Mfinanga S, Kivuyo S, Rugemalila J, Segal ES, Needleman L, Molloy SF, Kwon-Chung J, et al.. 2019. Dynamic ploidy changes drive fluconazole resistance in human cryptococcal meningitis. J Clin Invest 129:999–1014. doi:10.1172/JCI124516 30688656 PMC6391087

[48] Basso LR Jr, Gast CE, Bruzual I, Wong B. 2015. Identification and properties of plasma membrane azole efflux pumps from the pathogenic fungi *Cryptococcus gattii* and *Cryptococcus neoformans*. J Antimicrob Chemother 70:1396–1407. doi:10.1093/jac/dku554 25630649 PMC4398472

[49] Chang M, Sionov E, Khanal Lamichhane A, Kwon-Chung KJ, Chang YC. 2018. Roles of three *Cryptococcus neoformans* and *Cryptococcus gattii* Efflux pump-coding genes in response to drug treatment. Antimicrob Agents Chemother 62:e01752–17. doi:10.1128/AAC.01751-17 PMC5913978 29378705

[50] Sionov E, Lee H, Chang YC, Kwon-Chung KJ. 2010. *Cryptococcus neoformans* overcomes stress of azole drugs by formation of disomy in specific multiple chromosomes. PLoS Pathog 6:e1000848. doi:10.1371/journal.ppat.1000848 20368972 PMC2848560

[51] Singulani J de L, Oliveira LT, Ramos MD, Fregonezi NF, Gomes PC, Galeane MC, Palma MS, Fusco Almeida AM, Mendes Giannini MJS. 2021. The antimicrobial peptide MK58911-NH_2_ acts on planktonic, biofilm, and intramacrophage cells of *Cryptococcus neoformans*. Antimicrob Agents Chemother 65:e0090421. doi:10.1128/AAC.00904-21 34516241 PMC8597745

[52] Oliveira NK, Bhattacharya S, Gambhir R, Joshi M, Fries BC. 2022. Novel ABC transporter associated with fluconazole resistance in aging of *Cryptococcus neoformans*. JoF 8:677. doi:10.3390/jof8070677 35887434 PMC9320417

[53] Winski CJ, Qian Y, Mobashery S, Santiago-Tirado FH. 2022. An atypical ABC transporter is involved in antifungal resistance and host interactions in the pathogenic fungus *Cryptococcus neoformans*. mBio 13:e0153922. doi:10.1128/mbio.01539-22 35726920 PMC9426558

[54] Cowen LE, Steinbach WJ. 2008. Stress, drugs, and evolution: the role of cellular signaling in fungal drug resistance. Eukaryot Cell 7:747–764. doi:10.1128/EC.00041-08 18375617 PMC2394973

[55] Onyewu C, Blankenship JR, Del Poeta M, Heitman J. 2003. Ergosterol biosynthesis inhibitors become fungicidal when combined with calcineurin inhibitors against *Candida albicans*, *Candida glabrata*, and *Candida krusei*. Antimicrob Agents Chemother 47:956–964. doi:10.1128/AAC.47.3.956-964.2003 12604527 PMC149324

[56] Schuetzer-Muehlbauer M, Willinger B, Egner R, Ecker G, Kuchler K. 2003. Reversal of antifungal resistance mediated by ABC efflux pumps from *Candida albicans* functionally expressed in yeast. Int J Antimicrob Agents 22:291–300. doi:10.1016/s0924-8579(03)00213-9 13678837

[57] Maesaki S, Marichal P, Hossain MA, Sanglard D, Vanden Bossche H, Kohno S. 1998. Synergic effects of tactolimus and azole antifungal agents against azole-resistant *Candida albican* strains. J Antimicrob Chemother 42:747–753. doi:10.1093/jac/42.6.747 10052898

[58] Del Poeta M, Cruz MC, Cardenas ME, Perfect JR, Heitman J. 2000. Synergistic antifungal activities of bafilomycin A(1), fluconazole, and the pneumocandin MK-0991/caspofungin acetate (L-743,873) with calcineurin inhibitors FK506 and L-685,818 against *Cryptococcus neoformans*. Antimicrob Agents Chemother 44:739–746. doi:10.1128/AAC.44.3.739-746.2000 10681348 PMC89756

[59] Khunweeraphong N, Kuchler K. 2021. Multidrug resistance in mammals and fungi-from MDR to PDR: a rocky road from atomic structures to transport mechanisms. Int J Mol Sci 22:4806. doi:10.3390/ijms22094806 33946618 PMC8124828

[60] Rossato L, Venturini TP, de Azevedo MI, Santurio JM, Alves SH. 2022. *In vitro* activity of immunosuppressive agents against *Cryptococcus neoformans*. Enferm Infecc Microbiol Clin (Engl Ed) 40:86–88. doi:10.1016/j.eimce.2020.09.007 35120653

[61] Medina-Alarcón KP, Singulani JL, Voltan AR, Sardi JCO, Petrônio MS, Santos MB, Polaquini CR, Regasini LO, Bolzani VS, da Silva DHS, et al.. 2017. Alkyl protocatechuate-loaded nanostructured lipid systems as a treatment strategy for *Paracoccidioides brasiliensis* and *Paracoccidioides lutzii in vitro*. Front Microbiol 8:2310. doi:10.3389/fmicb.2017.01048 28659880 PMC5466964

[62] Yuan C, Wang Y, Zhang L, Wang D. 2024. Procatechuic acid and protocatechuic aldehyde increase survival of *Caenorhabditis elegans* after fungal infection and inhibit fungal virulence. Front Pharmacol 15:1338543. doi:10.3389/fphar.2024.1396733 PMC11150623 38841375

[63] Merkl R, Hrádková I, Filip V, Šmidrkal J. 2010. Antimicrobial and antioxidant properties of phenolic acids alkyl esters. Czech J Food Sci 28:275–279. doi:10.17221/132/2010-CJFS

[64] Kubo I. 2003. Molecular design of multifunctional antibacterial agents against methicillin resistant *Staphylococcus aureus* (MRSA). Bioorganic & Medicinal Chemistry 11:4255–4262. doi:10.1016/S0968-0896(03)00433-4 12951156

[65] Costa-Orlandi CB, Bila NM, Bonatti JLC, Vaso CO, Santos MB, Polaquini CR, Santoni Biasioli MM, Herculano RD, Regasini LO, Fusco-Almeida AM, et al.. 2023. Membranolytic activity profile of Nonyl 3,4-dihydroxybenzoate: a new anti-biofilm compound for the treatment of dermatophytosis. Pharmaceutics 15:1402. doi:10.3390/pharmaceutics15051402 37242644 PMC10223064

[66] Rossi SA. 2014. Estudo dos mecanismos de resistência e virûlência de isolados de *Cryptococcus neoformans* e *Cryptococcus gattii* e quantificação de genes de bomba de efluxo pós-tratamento com galatos de alquila PhD thesis, Universidade Estadual Paulista, Araraquara, Brazil

[67] Holmes AR, Cardno TS, Strouse JJ, Ivnitski-Steele I, Keniya MV, Lackovic K, Monk BC, Sklar LA, Cannon RD. 2016. Targeting efflux pumps to overcome antifungal drug resistance. Future Med Chem 8:1485–1501. doi:10.4155/fmc-2016-0050 27463566 PMC5827819

[68] Hajiagha MN, Kafil HS. 2023. Efflux pumps and microbial biofilm formation. Infect Genet Evol 112:105459. doi:10.1016/j.meegid.2023.105459 37271271

[69] Váchová L, Stovícek V, Hlavácek O, Chernyavskiy O, Stĕpánek L, Kubínová L, Palková Z. 2011. Flo11p, drug efflux pumps, and the extracellular matrix cooperate to form biofilm yeast colonies. J Cell Biol 194:679–687. doi:10.1083/jcb.201103129 21875945 PMC3171128

[70] Li P, Seneviratne CJ, Alpi E, Vizcaino JA, Jin L. 2015. Delicate metabolic control and coordinated stress response critically determine antifungal tolerance of *Candida albicans* biofilm persisters. Antimicrob Agents Chemother 59:6101–6112. doi:10.1128/AAC.00543-15 26195524 PMC4576078

[71] Askew C, Sellam A, Epp E, Hogues H, Mullick A, Nantel A, Whiteway M. 2009. Transcriptional regulation of carbohydrate metabolism in the human pathogen *Candida albicans*. PLoS Pathog 5:e1000612. doi:10.1371/journal.ppat.1000612 19816560 PMC2749448

[72] Brauner A, Fridman O, Gefen O, Balaban NQ. 2016. Distinguishing between resistance, tolerance and persistence to antibiotic treatment. Nat Rev Microbiol 14:320–330. doi:10.1038/nrmicro.2016.34 27080241

[73] Brucker K, Cremer K, Cammue BPA, Thevissen K. 2016. Protocol for determination of the persister subpopulation in *Candida albicans* biofilms, p 61–72. *In* Michiels J, Fauvart M (ed), Bacterial persistence: methods in molecular biology. Humana Press, New York, NY. 10.1007/978-1-4939-2854-5_6 26468100

[74] Sun J, Li Z, Chu H, Guo J, Jiang G, Qi Q. 2016. *Candida albicans* amphotericin b-tolerant persister formation is closely related to surface adhesion. Mycopathologia 181:41–49. doi:10.1007/s11046-015-9894-1 26381156

[75] Vincent BM, Lancaster AK, Scherz-Shouval R, Whitesell L, Lindquist S. 2013. Fitness trade-offs restrict the evolution of resistance to amphotericin B. PLoS Biol 11:e1001692. doi:10.1371/journal.pbio.1001692 24204207 PMC3812114

[76] Rosenberg A, Ene IV, Bibi M, Zakin S, Segal ES, Ziv N, Dahan AM, Colombo AL, Bennett RJ, Berman J. 2018. Antifungal tolerance is a subpopulation effect distinct from resistance and is associated with persistent candidemia. Nat Commun 9:2470. doi:10.1038/s41467-018-04926-x 29941885 PMC6018213

[77] Melhem MSC, Leite Júnior DP, Takahashi JPF, Macioni MB, Oliveira L de, de Araújo LS, Fava WS, Bonfietti LX, Paniago AMM, Venturini J, et al.. 2024. Antifungal resistance in cryptococcal infections. Pathogens 13:128. doi:10.3390/pathogens13020128 38392866 PMC10891860

[78] Bongomin F, Oladele RO, Gago S, Moore CB, Richardson MD. 2018. A systematic review of fluconazole resistance in clinical isolates of *Cryptococcus* species. Mycoses 61:290–297. doi:10.1111/myc.12747 29377368

[79] Ke W, Xie Y, Chen Y, Ding H, Ye L, Qiu H, Li H, Zhang L, Chen L, Tian X, et al.. 2024. Fungicide-tolerant persister formation during cryptococcal pulmonary infection. Cell Host & Microbe 32:276–289. doi:10.1016/j.chom.2023.12.012 38215741

[80] Zhao Y, Ye L, Zhao F, Zhang L, Lu Z, Chu T, Wang S, Liu Z, Sun Y, Chen M, et al.. 2023. *Cryptococcus neoformans*, a global threat to human health. Infect Dis Poverty 12:20. doi:10.1186/s40249-023-01073-4 36932414 PMC10020775

[81] Shourian M, Qureshi ST. 2019. Resistance and tolerance to cryptococcal infection: an intricate balance that controls the development of disease. Front Immunol 10:66. doi:10.3389/fimmu.2019.00066 30761136 PMC6361814

[82] Uppuluri P, Nett J, Heitman J, Andes D. 2008. Synergistic effect of calcineurin inhibitors and fluconazole against *Candida albicans* biofilms. Antimicrob Agents Chemother 52:1127–1132. doi:10.1128/AAC.01397-07 18180354 PMC2258509

